# Effects of *unpaired 1* gene overexpression on the lifespan of *Drosophila melanogaster*

**DOI:** 10.1186/s12918-019-0687-x

**Published:** 2019-03-05

**Authors:** Alexey Moskalev, Ekaterina Proshkina, Alex Zhavoronkov, Mikhail Shaposhnikov

**Affiliations:** 1 Engelhardt Institute of Molecular Biology, Russian Academy of Sciences, Moscow, 119991 Russia; 2 Institute of Biology, Komi Scientific Center, Ural Division, Russian Academy of Sciences, Syktyvkar, 167982 Russia; 3 Moscow Institute of Physics and Technology, Dolgoprudny, 141700 Russia; 4 Insilico Medicine, Rockville, MD USA

**Keywords:** Lifespan, *Drosophila melanogaster*, JAK/STAT signaling pathway, Unpaired ligand, Mifepristone

## Abstract

**Background:**

The JAK/STAT signaling pathway is involved in many aging-related cellular functions. However, effects of overexpression of genes controlling JAK/STAT signal transduction on longevity of model organisms have not been studied. Here we evaluate the effect of overexpression of the *unpaired 1* (*upd1*) gene, which encodes an activating ligand for JAK/STAT pathway, on the lifespan of *Drosophila melanogaster*.

**Results:**

Overexpression of *upd1* in the intestine caused a pronounced shortening of the median lifespan by 54.1–18.9%, and the age of 90% mortality by 40.9–19.1% in males and females, respectively. In fat body and in nervous system of male flies, an induction of *upd1* overexpression increased the age of 90% mortality and median lifespan, respectively. An increase in *upd1* expression enhanced mRNA levels of the JAK/STAT target genes *domeless* and *Socs36E*.

**Conclusions:**

Conditional overexpression of *upd1* in different tissues of *Drosophila* imago induces pro-aging or pro-longevity effects in tissue-dependent manner. The effects of *upd1* overexpression on lifespan are accompanied by the transcription activation of genes for the components of JAK/STAT pathway.

## Background

The JAK/STAT (Janus kinase/signal transducers and activators of transcription) signaling pathway is involved in many aging-related functions, including cell proliferation, differentiation, survival, apoptosis, cell senescence, state of heterochromatin, and expression of a variety of genes [1–6]. In addition, the JAK/STAT pathway coordinates processes of stem cell proliferation and differentiation in *Drosophila* intestine [7–9], Malpighian tubules [10], and testis niche [11], thereby establishing tissue homeostasis. JAK/STAT signaling is an important component of the conserved transcriptional network driving myogenesis [12]. The expression levels of JAK/STAT signal pathway proteins are variable throughout the organism lifespan, and tend to decline with age [13].

In *Drosophila melanogaster*, the *unpaired 1* (*upd1*) gene encodes the most potent ligand for the JAK/STAT signaling pathway [14, 15]. Earlier, Boyle et al. demonstrated that the expression of *upd* in the stem cells of male gonad niche decreases at the old age proportionally to a decline of stem cell population [16]. Overexpression of *upd* in gonads of old males impedes the degeneration of germ line stem cells [16]. Thus, literature data suggest a connection between the processes of aging and JAK/STAT signal pathway activity. At the same time, the effects of overexpression of genes, which encode cytokines controlling the JAK/STAT signaling pathway, on the lifespan have not been studied.

The purpose of the present work is to elucidate whether conditional overexpression of *upd1*in specific tissues (intestine, fat body, and nervous system) affects longevity of *Drosophila melanogaster* imago.

## Results

### Effects of the *upd1* gene overexpression on the lifespan

In order to analyze the lifespan effect of the overexpression of cytokines that control JAK/STAT signaling, we conditionally activated the overexpression of the *upd1* gene in the intestine, fat body, and nervous system throughout the imago stage.

**Fig. 1:**
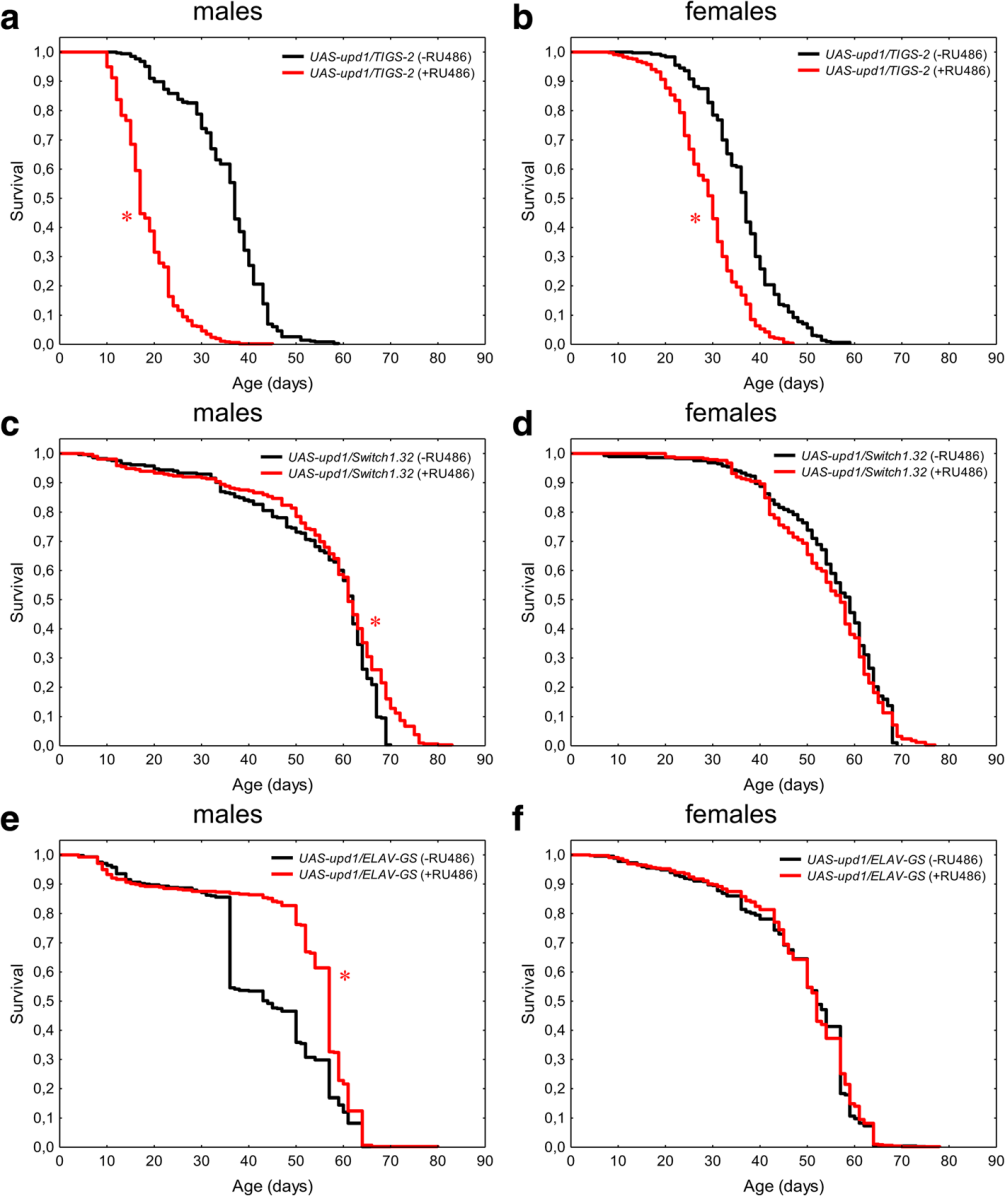
Effects of the *upd1* gene overexpression in the intestines (**a**, **b**), fat body (**c**, **d**) and nervous system (**e**, **f**) on the lifespan of *Drosophila melanogaster* males (**a**, **c**, **e**) and females (**b**, **d**, **f**). The results of two experimental replications are merged. *p < 0.001, Kolmogorov–Smirnov test. The presented data correspond to the Table 1

Overexpression of *upd1* in the fly intestine caused a statistically significant decrease in the lifespan of both sexes (*p* < 0.001) (Table 1, Fig. 1a and b). In males and females, the median lifespan was reduced by 54.1 and 18.9%; the age of 90% mortality decreased by 40.9 and 19.1%, respectively. Males and females with *upd1* overexpression in intestinal cells exhibited higher rates of initial (*R*_0_) and age-dependent (α) mortality (Table 1).

**Table 1:** Effects of conditional (RU486-induced) *upd1* gene overexpression in intestinal cells (*TIGS-2*), fat body cells (*Switch1.32*) and nervous system cells (*ELAV-GS*) on the lifespan of imago *Drosophila melanogaster*

Genotype	Variant	Sex	M	dM	Cox-Mantel test	90%	d90%	Wang–Allison test	MRDT	dMRDT	α	R_0_	n
*UAS-upd/TIGS-2*	-RU486	♂	37			44			5.5		0.12519	0.00094	345
*UAS-upd/TIGS-2*	+RU486	♂	17	−54.1	p < 0.001	26	−40.9	p < 0.001	5.5	−0.3	0.12555	0.00934	454
*UAS-upd/TIGS-2*	-RU486	♀	37			47			5.6		0.12277	0.00087	423
*UAS-upd/TIGS-2*	+RU486	♀	30	−18.9	p < 0.001	38	−19.1	p < 0.001	4.9	−12.4	0.14013	0.0015	470
*UAS-upd/Switch1.32*	-RU486	♂	62			67			6.3		0.10991	0.00013	283
*UAS-upd/Switch1.32*	+RU486	♂	61	−1.6	p > 0.05	72	7.5	p < 0.0001	7.3	16.4	0.09445	0.00023	312
*UAS-upd/Switch1.32*	-RU486	♀	59			68			5.6		0.12379	0.00007	283
*UAS-upd/Switch1.32*	+RU486	♀	57	−3.4	p > 0.05	68	0.0	p > 0.05	6.5	15.9	0.10679	0.00019	336
*UAS-upd/ELAV-GS*	-RU486	♂	43.5			61			8.9		0.07795	0.0016	449
*UAS-upd/ELAV-GS*	+RU486	♂	57	31.0	p < 0.001	64	4.9	p > 0.05	6.0	−32.1	0.11475	0.00016	417
*UAS-upd/ELAV-GS*	-RU486	♀	52			60			6.5		0.10705	0.00033	448
*UAS-upd/ELAV-GS*	+RU486	♀	52	0.0	p > 0.05	61	1.7	p > 0.05	6.4	−0.7	0.10776	0.00031	462

RU486 – control, without mifepristone; +RU486 – overexpression, with mifepristone; ♂ – males; ♀ – females; М – (days); 90% – age of 90% mortality (days); MRDT – mortality rate doubling time (days); α and R_0_ – Gompertz equation parameters; n – sample size. dМ, d90%, dMRDT – the differences in median lifespan, age of 90% mortality and MRDT estimated between -RU486 and + RU486 variants (%)

In males and females, conditional overexpression of the *upd1* gene in fat body cells (Table 1, Fig. 1c and d) did not produce a statistically significant effect on the median lifespan (*p* > 0.05). At the same time, males demonstrated an increase of 90% mortality age (by 7.5%, *p* < 0.0001). In flies of both sexes, a slight decrease of the age-dependent mortality rate was detected (Table 1).

Conditional activation of *upd1* overexpression in nervous system cells (Table 1, Fig. 1e and f) caused altered survival measures. Neuron-specific overexpression of *upd1* led to a statistically significant increase of the median lifespan (by 31%, *p* < 0.001) in males only. However, the effect of the median lifespan enhancement was accompanied by a decrease in mortality rate doubling time (MRDT) (by 32.1%) and accelerated aging (Table 1).

### Quantification of the *upd1*, *dome*, and *Socs36E* genes expression in different tissues

**Fig. 2:**
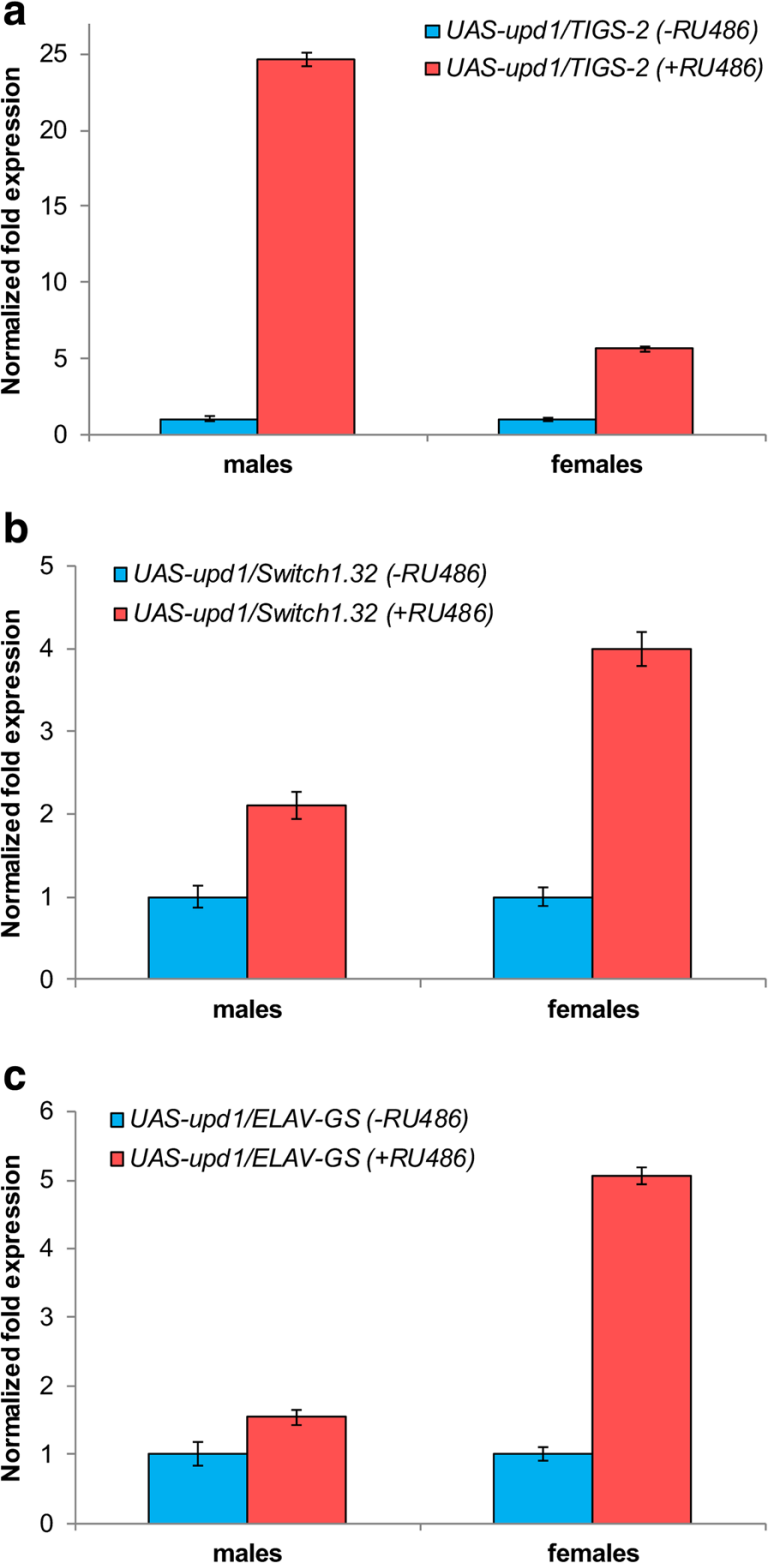
Relative expression levels of the *upd1* gene in *Drosophila melanogaster* imago tissues activated by tissue-specific drivers: **a** intestines (*TIGS-2*); **b** fat body (*Switch1.32*); **c** nervous system (*ELAV-GS*)

Analysis of relative expression levels in different imago tissues using the qRT-PCR method confirmed activation of the *upd1* gene expression in response to mifepristone treatment (Fig. 2). In male intestinal cells, expression of the *upd1* gene was enhanced 25-fold; in females, 6-fold (Fig. 2a). In fat body cells, expression of the *upd1* gene increased 2-fold in males and 4-fold in females (Fig. 2b). In nervous system cells, a 1.5-fold enhancement of expression was observed in males, and a 5-fold increase in females (Fig. 2c).

**Fig. 3:**
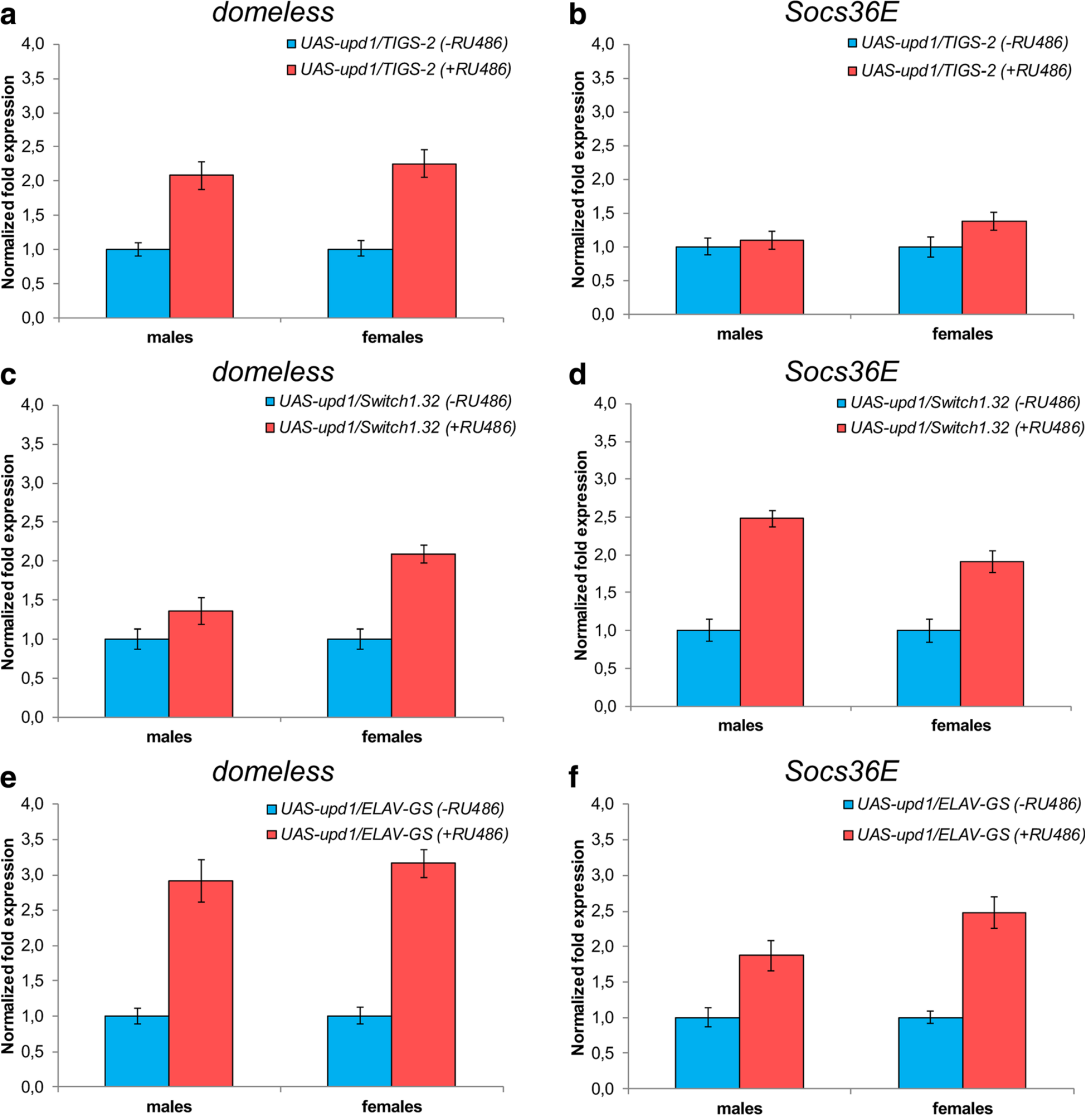
Relative expression levels of the *domeless* and *Socs36E* in *Drosophila melanogaster* imago tissues (**a**, **b** intestines (*TIGS-2*), **c**, **d** fat body (*Switch1.32*), and **e**, **f** nervous system (*ELAV-GS*)) with overexpression of *upd1*

To clarify whether the lifespan alteration in flies overexpressing *upd1* is associated with activation of the JAK/STAT signaling pathway, we analyzed the expression level of *domeless* and *Suppressor of cytokine signaling at 36E* (*Socs36E*), which are the JAK/STAT target genes [17, 18]. We found that the activation of *upd1* expression led to a 1.5–3-fold increase in the levels of *domeless* in all studied tissues, and a 1.5–2.5-fold increase in the levels of *Socs36E* mRNA in the fat body and nervous system, respectively (Fig. 3). These data suggest that overexpression of *upd1* transcription is accompanied by the induction of JAK/STAT signaling components and may influence the *Drosophila* lifespan through this pathway.

### Histology of intestine

**Fig. 4:**
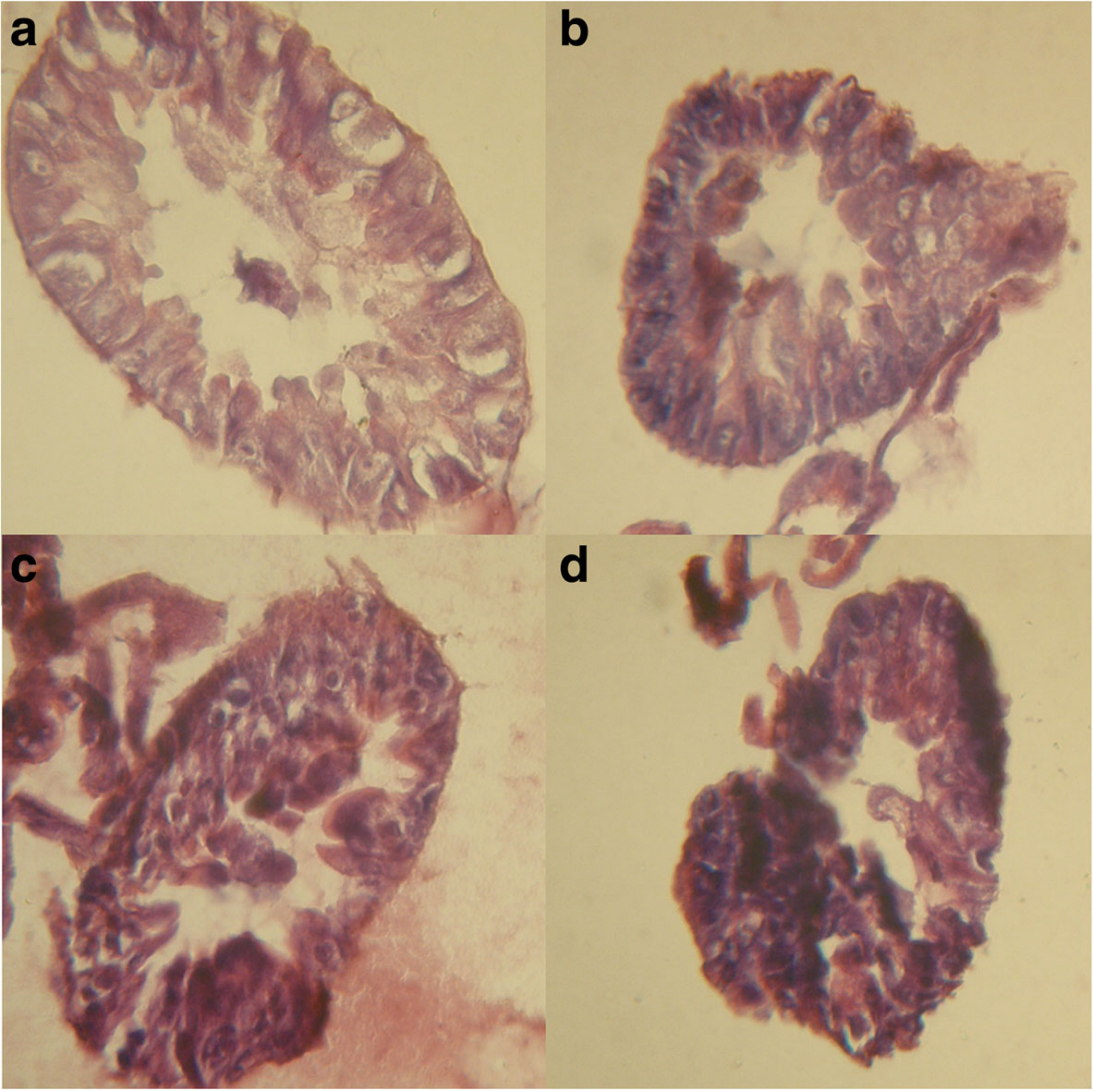
Mifepristone induces dysplasia of gut epithelium in in *UAS-upd1*/*TIGS-2* females. Transverse sections through the intestine of: **a** 1 day old (-RU486), **b** 10 days old (-RU486), **c** 1 day old (+RU486), **d** 10 days old (+RU486). All magnitudes × 800

To find whether the lifespan decreasing in *UAS-upd1*/*TIGS-2* flies within the digestive system is caused by disruption of intestinal tissue homeostasis, we carried out the histological study. The histological observation of intestines revealed that the mifepristone treatment induced progressive dysplasia of gut epithelium in *UAS-upd1*/*TIGS-2* females at ages from 1 to 10 days (Fig. 4). This disruption of intestinal tissue homeostasis may result in the increasing mortality rates of flies with *upd1* overexpression in intestine.

## Discussion

The JAK/STAT pathway is one of the few signaling cascades that are evolutionarily conserved in multicellular animals from flies to humans both on the structural and functional levels [1, 19]. JAK/STAT signaling is involved in the transduction of intercellular biochemical signals essential for the development and the maintenance of homeostasis [19]. In mammals, the JAK/STAT pathway is activated by a broad spectrum of cytokines and growth factors, which stimulate cell proliferation, differentiation, migration, and apoptosis [19]. These cellular processes are critical for hematopoiesis, immune development, adipogenesis, sexually dimorphic growth, and aging. The expression levels of these signal pathway proteins do fluctuate throughout the lifespan of an organism, but decrease with age in macrophages, natural killer cells, hippocampus, and myocardium [13].

In contrast to mammals, *Drosophila melanogaster* genome encodes for only three ligands with JAK/STAT activating properties. These ligands are encoded by the *unpaired* gene family that includes *upd1, upd2,* and *upd3* [15]. The reduced expression of *upd1* in old *Drosophila* males correlates with an overall decrease of germ line stem cells number [16]. Thus, data obtained for mammals and flies point towards alterations of the JAK/STAT signaling activity in aging cells and organs. However, the effect of the JAK/STAT signal pathway overactivation on the organism lifespan was not studied previously. At the same time, a number of recent publications demonstrates lifespan extension in flies with overexpression of genes, which enhance resistance to oxidative stress (*SOD1*) [20] and survival under stress conditions (*FOXO*) [21, 22], control repair of proteins (*PCMT* and *dmsrA*) [23, 24] and DNA repair [25], increase female fecundity at late ages (*hebe* and *magu*) [26], and reduce insulin/IGF-1 (UCP) [27] or TOR kinase (*dTsc1*, *dTsc2* or dominant-negative forms of *dTOR* or *dS6K*) [28] signaling pathways. Loss of function and knockdown of genes regulating the insulin/IGF-1 signaling pathway during growth (*Lnk*) [29], energy metabolism (*Indy* and mitochondrial electron transport chain genes) [30, 31], or affecting intracellular signaling pathways (*mth*) [32] extend *Drosophila* lifespan, as well.

The crucial tissues for the most of longevity genes activity are intestinal [30], adipose [22, 28], and nervous [20, 24, 27, 31] tissues, which are the principal sites of metabolism in the fly.

Thus, in order to investigate the effect of the JAK/STAT signal pathway overactivation on the organism lifespan, we activated overexpression of the *upd1* gene that is the most potent ligand for the JAK/STAT signaling pathway [15] in *Drosophila melanogaster* in some indispensable-to-longevity imago tissues (intestine, fat body, nervous system). The use of conditional mifepristone-activated tissue-specific Gene-Switch drivers enables us to control not only the tissue, but also the stage of transgene expression induction (throughout the imago lifespan), as well as to exclude the effects of different genetic backgrounds on the lifespan [33]. However mifepristone has been reported to affect lifespan of males and mated females differently. It increases the longevity of mated females but not males by various mechanisms including modulation of feeding rate and innate immune response [34–36]. Sex-specific differences in the response to mifepristone treatment may explain the different effects of the *upd1* overexpression in the fat body and the nervous system in males and females. The positive effects of mifepristone feeding and *upd1* overexpression in these tissues are indistinguishable in mated females. At the same time, the strongly pronounced negative effect of the *upd1* overexpression in the intestine on lifespan does not overlap with the positive effect of mifepristone.

In addition the Gene-Switch drivers which were used in our study has been reported to demonstrate sex-specific differences in the expression levels (both temporal and spatial) in the presence of the inducer and leaky expression in non-target tissues in the absence of the inducer [37], which makes any meaningful comparisons between the males and females very complicated.

As shown by a series of studies, intestines of old flies and flies exposed to stress exhibit depletion of epithelium accompanied by excessive proliferation and aberrant differentiation of stem cells [38, 39]. Moderate activation of JNK signaling in intestinal cells in response to stress results in differentiation anomalies and in substantial decrease of a lifespan [38]. Maintaining of tissue homeostasis involves the JAK/STAT signaling pathway, too [7].

We have shown that induction of *upd1* overexpression in the intestine causes significant lifespan shortening in individuals of both sexes. Accelerated aging of the organism is seen upon both strong induction of *upd1* expression in males (25-fold), moderate induction in females (6-fold), and weak activation of the *domeless* gene (2-fold). This effect could be caused by disruption of intestinal tissue homeostasis resulting from excessive activation of JAK/STAT. As it was shown by Li et al., chronic activation of JAK/STAT signaling in the gut of aging *Drosophila* is associated with age-related metaplasia, commensal dysbiosis and functional decline of the gastrointestinal tract. Accordingly, inhibition of JAK/STAT signaling in the stomach-like copper cell region prevents these negative changes and extends lifespan of flies [40]. Histological observations of female intestines display that *upd1* overexpression results in dysplasia of gut epithelium, which may be the cause of lifespan decrease to a considerable extent.

Agaisse et al. showed that activation of JAK/STAT pathway in the *Drosophila* fat body by upd3 ligand facilitates the formation of immune response to septic shock [41]. It is also known that JAK/STAT signaling demonstrates anti-apoptotic activity [42]. An increase of apoptosis levels in postmitotic tissues with age can lead to a decline of functional activity of the tissue as a result of irretrievable cell loss. Previously, Zheng et al. have shown that under normal conditions, fly aging is accompanied by a steady increase of apoptosis level in muscle and fat cells [43]. Therefore, an increase of the age of 90% mortality in male individuals at weak level (2-fold) of *upd1* overexpression in the fat body may be caused by activation of the immune system and anti-apoptotic activity.

It is of note that sustained production of macrophage-derived upd3 in response to chronic lipid-rich diet was associated with activation of JAK-STAT signaling, increased fat storage, reduced insulin sensitivity, hyperglycemia, and a shorter lifespan of *Drosophila* flies [44]. Upd2 is produced by the *Drosophila* fat body in response to dietary fats and sugars and promote secretion of *Drosophila* insulin-like peptides [45]. The opposite effects of different unpaired ligands on the lifespan and aging may be associated with their different functions.

We also demonstrated that the low level of *upd1* overexpression (1.5-fold) in the nervous system of males was accompanied by the induction of JAK/STAT signaling components *domeless* (3-fold) and *Socs36E* (2-fold) and led to increase of median lifespan. As it was shown by Chiba et al., age-dependent deterioration in the JAK2/STAT3 axis plays a critical role in the pathogenesis of Alzheimer’s disease [46]. The activity of the JAK/STAT pathway has been shown to be involved in synaptic plasticity in the mammalian brain [47]. Therefore, the overexpression of *upd1* and activation of the JAK/STAT pathway can potentially prevent neurodegeneration and age-related deficits in synaptic plasticity contributing to longevity.

## Conclusions

Thus, conditional overexpression of the *upd1* gene encoding a JAK/STAT signaling ligand results in alteration of the *Drosophila* lifespan depending on the tissue specificity of overexpression. The effects of *upd1* overexpression on lifespan are accompanied by the transcription activation of the JAK/STAT target genes *domeless* and *Socs36E*. As the JAK/STAT pathway is evolutionarily conserved it may be possible to discover compounds that fit the criteria of geroprotector [48]. In our prior work we explored the effects of a variety of interventions on the *Drosophila* lifespan [49–53]. The new pathway analysis algorithms enable us to identify the pharmacological interventions for modulation of the levels of expression of individual genes and networks. In our future work we plan to test the compounds from DrugAge [54] and Geroprotectors.org [55] and other libraries potentially modulating *upd*, *domeless* and *Socs36E* on the lifespan of *Drosophila* and other organisms.

## Material and methods

### *Drosophila melanogaster* lines

*UAS-upd1* (genotype: *w*^1118^; *UAS-upd1*/*CyO*): transformed line in which expression of an additional copy of the *unpaired 1* gene is controlled by *UAS* promoter (kindly provided by Dr. James Castelli-Gair Hombría, Universidad Pablo de Olavide, Sevilla, Spain) [56].

*TIGS-2*: Gene-Switch driver line containing mifepristone-inducible GAL4 in intestine cells (kindly provided by Dr. Laurant Seroude, Queen’s University, Kingston, Canada) [37].

*Switch1.32* (genotype: *w*^1118^; P{*w*^+mW.hs^ = Switch1} *bun*^Switch1.32^/*CyO*): driver line containing mifepristone-inducible GAL4 in imago head fat body cells (kindly provided by Dr. Marc Tatar, Brown University, Providence, Rhode Island, USA) [33].

*ELAV-GS* (genotype: *y w; P{ELAV-GeneSwitch}*): Gene-Switch driver line containing mifepristone-inducible GAL4 in nervous system cells (kindly provided by Dr. Haig Keshishian, Yale University, New Haven, Connecticut, USA) [33].

### Activation of the *upd1* gene overexpression

Overexpression of the *upd1* gene in *Drosophila melanogaster* was activated using the GAL4/UAS system. Crossing of *UAS-upd1* line females with Gene-Switch system driver line males (*Switch1.32*, *ELAV-GS*, and *TIGS-2*) resulted in *UAS-upd1*/*TIGS-2*, *UAS-upd1*/*Switch1.32*, and *UAS-upd1*/*ELAV-GS* genotypes in F_1_. After eclosion males and females were allowed to mate during the 24 h and then separated according to sex. Overexpression was conditionally activated by feeding the F_1_ imagoes with yeast paste supplemented with 25 μg/ml mifepristone (RU486, Sigma-Aldrich, USA). Flies were treated with RU486 throughout life of adults. Control animals were fed yeast paste without RU486.

### Lifespan analysis

Control and experimental flies were maintained at 25 ± 0.5 °C under a 12 h light/12 h dark cycle and at densities of 30 same sex and age flies per vial with sugar-yeast medium [57] covered with a yeast paste. Flies were transferred to fresh medium three times per week. Lifespan was analyzed daily, separately for males and females. Every variant of the experiment was analyzed in two replications. Survival functions were estimated using the Kaplan–Meier procedure and plotted as survival curves. Median lifespan and the age of 90% mortality (maximum lifespan) were calculated. The initial (R_0_) and age-dependent (α) mortality (parameters of the Gompertz equation (*μ*(*х*) = *R*_0_e^*αx*^)) and the mortality rate doubling time (MRDT = ln2/*α*) were estimated. Nonparametric methods were used for statistical processing of results. Comparison of survival functions was done using the modified Kolmogorov–Smirnov test [58]. The Mantel–Cox test [59] was used to evaluate the statistical significance of medial lifespan differences. To test the statistical significance of differences in age of 90% mortality (maximum lifespan), the Wang–Allison test was used [60]. Statistical analysis was carried out using *Statistica* version 6.1, StatSoft, Inc., R, version 2.15.1 (The R Foundation) and OASIS 2: Online Application for Survival Analysis 2 [61].

### Quantitative real-time PCR

To quantify the gene expression levels in different imago tissues, 50 flies were used for mRNA isolation and cDNA synthesis per each experimental variant. The flies were treated by mifepristone during 3–5 days after imago hatching. Expression levels in males and females were estimated separately. Fly heads were excised to estimate the levels of *Switch1.32* and *ELAV-GS* driven expression in head fat body or nervous system, respectively. Intestines were dissected for *TIGS-2* driven expression estimation in digestive system. The homogenization of isolated organs using the SilentCrusher S homogenizer (Heidolph Instruments, Germany) was followed by RNA extraction with TRIzol Reagent (Invitrogen, USA), according to the manufacturer’s protocol. To assure the absence of RNA samples contamination by DNA, one of the PCRs was performed using *β-Tubulin56D* (*βTub56D*) gene primers without reverse transcription. For cDNA synthesis, the Oligo(dT) primer (Invitrogen, USA) and SuperScript III First-Stand Synthesis System reverse transcriptase (Invitrogen, USA) were used according to the manufacturer’s protocol. Real-time PCR was carried out using 30 μl of reaction mixture containing SYBR Green PCR Master Mix (Applied Biosystems, USA) in 200-μl PCR tubes with primers listed in Table 2. According to pcrEfficiency estimation used primers have similar efficiencies for PCR amplification [62]. Real-time PCR reactions were performed on the nucleic acid analyzer ANK-32 (Institute of Analytical Instrumentation of the Russian Academy of Sciences, Russia). All real-time PCR assays were run using the following program: 1) denaturing at 95°С for 10 min., 2) denaturing at 95°С for 15 s, 3) annealing at 60°С for 30 s, 4) elongation at 60°С for 30 s, 5) stages 2–4 were repeated 50 times. Amplification was carried out in separate tubes for each gene. 4–8 measurements were performed per each experimental variant. *β-Tubulin56D* was used as the endogenous reference gene. The relative quantification of the *upd1*, *domeless*, and *Socs36E* expression was carried out according to the 2^-ΔΔС(T)^ method [63] and expressed as relative fold change normalized to *β-Tubulin56D*. Cycle threshold (C_T_) values were determined using the *ANK32* software package, version 1.1 (Institute of Analytical Instrumentation of the Russian Academy of Sciences, Russia). The C_T_ values used for the following analysis were not over than 28 cycles. ΔΔC_T_ was calculated as ΔΔC_T_ = ΔC_T_(overexpression of *upd1*) – ΔC_T_(no overexpression of *upd1*), and each value ΔC_T_ = C_T_(target gene) – C_T_(reference gene).

**Table 2:** Nucleotide sequences of primers used for quantitative real-time PCR

Gene	Forward primer (5′-3′)	Reverse primer (5′-3′)	Product length (in bp)	Expected efficiency
*upd1*	agacagccgtcaaccagac	gcttcaaacgcttgttcatc	141	> 2
*domeless*	tcgctacatgacaacgtgaccgat	acgcacgctaatctcgtacttggt	132	> 2
*Socs36E*	gggcaaacagaacccagaaaccaa	tccgagctgcattccaataggtga	189	> 2
*βTub56D*	gcaactccactgccatcc	cctgctcctcctcgaact	216	> 2

### Histology of intestine

Intestines were dissected and fixed in mixture of 85% ethanol / 4% formaldehyde / 5% acetic acid / 1% glutaraldehyde for 48 h. Then, fixed organs were paraffin embedded, sectioned, hematoxylin and eosin (H&E) stained, and analyzed by light microscopy [64]. Images of the intestine of young and 10-day old female flies were acquired and analyzed.

## Abbreviations

JAK/STAT: Janus kinase/signal transducers and activators of transcription
MRDT: Mortality rate doubling time
*Socs36E*: *Suppressor of cytokine signaling at 36E*
